# Is Mixed Practice More Effective than Physical Practice Alone for the Acquisition of Non-dominant Side Kicking Performance?

**DOI:** 10.3389/fpsyg.2016.01665

**Published:** 2016-10-25

**Authors:** Kylie A. Steel, Eathan Ellem

**Affiliations:** ^1^Science and Health, Human Movement Science, Western Sydney UniversityPenrith, NSW, Australia; ^2^The MARCS InstituteSydney, NSW, Australia

**Keywords:** football, instep-kick, mixed practice, self-modeling, soccer, video training

## Abstract

Perceiving and executing unfamiliar movements, such as left handed/footed movement skills in sports, places additional demands on the perceptual-cognitive system of players that may increase errors. The video self-modeling (VSM) method may provide an accessible solution to this issue, therefore the purpose of this study was to investigate the effect of the VSM method on the improvement of a non-preferred side kicking task. *N* = 28 participants engaged in one of three conditions; Mirror reversed/ physical practice (PP), best-of/ PP, or physical practice only. Though not significant, data analysis indicated improved kicking accuracy for all groups, with VSM groups showing the most improvement. However, qualitative data revealed the “best-of” group demonstrated more positive views toward their progress compared to other groups, and both VSM groups were more likely to attend to movement cues than target based cues. These trends may suggest merit for the use of VSM techniques, though its application and the source of mechanistic factors warrant further investigation.

## Introduction

Football (soccer) is a fast moving team sport with players required to manipulate a ball across a field and into the territory of the opposing team to score points. Effective use of the entire field provides more affordances, however these opportunities may be lost if the receiving player intercepts the ball with their non-preferred foot. Consequently, players must reorient their body position to use their preferred side which adds time for movement execution in addition to increasing the chance of errors. Further, when defending against players who are left handed, an unfamiliar situation is presented which challenges a player's perceptual-cognitive capacity that may result in decision-making errors. Despite the obvious disadvantages these issues can cause for players, coaches seldom include training that addresses bilateral movement execution, most likely due to the competing demands of other factors perceived to be more advantageous in games. However, a method that may provide a solution to this issue is observational learning and practice.

Observational learning is a technique that involves an individual watching the performance of another person (e.g., expert, novice, or oneself). The observation of others is useful, enabling the observer to view how a movement may be best performed, however it may also encourage the learner to mimic the movement patterns of another whose movement signatures and anthropometrics do not match their own. This may therefore result in frustration and a decrease in adherence for practicing the skill.

More recently, researchers have investigated video-self modeling (VSM) or observation of oneself performing various sports related movements, e.g., gymnastics (Winfrey and Weeks, 1993), running (Gonzales et al., 2015), weightlifting (Franks and Maile, 1991), swimming (Dowrick and Dove, 1980), Australian Rules Football (Steel et al., 2013a,b), and cycling (Jennings et al., 2013). Unlike findings in literacy (Rao et al., 2012; Gillies, 2013), autism (Bellini et al., 2007), cerebral palsy (Dowrick, 1976) and other behavioral sciences (Dowrick, 2012), sport movement studies have indicated mixed results in support of the learning efficacy of this method (e.g., Starek and McCullagh, 1999; Law and Ste-Marie, 2005; Feltz et al., 2008).

Edited observation of oneself has been implemented using a number of techniques. These include observers viewing the VSM footage of only their best performance, for instance the feedforward (FF) method (Steel et al., 2013a,b). FF is implemented by eliminating negative aspects of one's performance, or in more recent examples reversing performances of the dominant hand or foot to show apparent future performances of a skill (Steel et al., 2013a,b).

Dowrick (1976) employed this technique for a young girl with cerebral palsy who was unable to step up on sidewalks or step over obstacles. Using relatively crude video editing techniques, Dorwick “spliced” together a sequence whereby she appeared to successfully complete a circuit full of these obstacles with minimal hesitation. After several observations of the video the girl was more able to walk over these obstacles without hesitation. Observation of this future “successful” performance may have provided her with the belief that she was able to complete the task (Winfrey and Weeks, 1993).

In sports, the FF method has been employed to improve the non-dominant side kick distance and accuracy of Australian Rules (AR) football players (Steel et al., 2013a). In an initial case study approach, participants were required to kick toward a target 50 m away that was 6.4 m in width (standard ARs goal post width). Footage of their best dominant foot performances were then reversed and recorded to a personalized DVD which participants observed three times a day for 14 days. Data analysis demonstrated players' improved side-to-side kick performance from pre- to post-test, which continued at the retention test. A subsequent study involving a larger sample group provided further evidence of the effectiveness of VSM techniques (Steel et al., 2013b), where Australian Rules football players were asked to complete a series of dominant and non-dominant side kicks toward a target 25 m away as accurately as possible. Pre- and post-data comparisons demonstrated improvement on the non-dominant side. However, after a 3 week retention period, performance had decreased to below that of initial performance. Participant questioning after the retention test suggested they had realized the footage was not of their non-dominant foot, which was not disclosed during the study. This may have resulted in participants questioning the belief they had developed their new ability.

As interest in observational learning and practice continues with techniques such as VSM, being extended to include a physical practice component (Ong et al., 2012). Research suggests VSM is effective; however improvements are greater when coupled with physical practice (Ong et al., 2012). These studies have used various motor skills to investigate mixed practice, however there is a paucity of research regarding its use for the improvement of the non-preferred side, despite significant advantages in sport.

Findings in this evolving area of research provide sufficient evidence that VSM methods have promise for motor skill acquisition; therefore the purpose of this study was to investigate the effectiveness of the FF approach and possible factors that might mediate its success in improving a non-preferred side skill. Specifically we investigated the effect different practice conditions had on the performance of a non-preferred side kicking task. We hypothesized that VSM (mirror reversed or best-of) in conjunction with physical practice would be more effective than physical practice only. Secondly, we propose due to the more fluid nature of the technique used on the preferred foot, 'mirror reversed' VSM would be more effective than “best-of” examples using the best of the non-preferred foot. We also suggest VSM would increase a participant's perception of non-preferred side ability, and finally the preferred foot performance would also improve with VSM training based on bilateral transfer of learning principles.

## Methods

### Participants

Thirty three female student and faculty members of the university aged 19–42 years (*M*_age_ = 25.4 ± 6.6) volunteered to take part in the study. Thirty participants self-reported their right foot was dominant when participating in motor skill tasks, with self-reporting left-footed participants (*n* = 3) excluded to ensure consistency within the sample group. Two additional participants did not return for training or post-testing due to illness or other commitments, thus the final sample was *N* = 28 (*M*_age_ = 25.7 ± 7.5). Mean experience (years) in kicking sports was assessed via a kicking questionnaire (*M*_experience_ = 3.3 ± 5.4). Ethics approval for the study was obtained from the University Human Research Ethics Committee and participants gave written informed consent prior to commencing the study (H9243). The study conforms to relevant regulatory conditions and participants were randomly allocated to one of three video-training groups: (1) Mirror reversed (VSM-m) (*n* = 9) (*M*_age_ = 26.3 ± 4.8; *M*_experience_ = 3 ± 4.6), (2) Best-of (VSM-b) (*n* = 10) (*M*_age_ = 24.1 ± 1.4; *M*_experience_ = 3.1 ± 4.9), (3) Physical practice only (PP-only) (*n* = 9) (*M*_age_ = 25.5 ± 7.2; *M*_experience_ = 3.3 ± 7.2).

### Procedure

#### Pre-testing

Participants completed three sessions within this study. The first session consisted of the pre-test and collection of basic demographic information including: age, footedness (self-report), and a kicking experience questionnaire. Participants then completed a 20 kick warm-up period, where they were allowed to kick the ball to the target using either foot. The pre-test consisted of the completion of 26 soccer (instep) kicks with both the dominant and non-dominant foot (13 each side; See Supplementary Video File). The order of kicks was randomized across participants in order to minimize learning effects. During the pre-test, participants were filmed from behind and slightly to the side using a SONY digital video camera at 25 fps. This video footage was then used to create practice training DVDs and showed kinematic data (markers located on ankle, knee, hip, wrist, elbow, and shoulder), ball path, and scoring result to the participant.

#### Scoring

**Figure 1:**
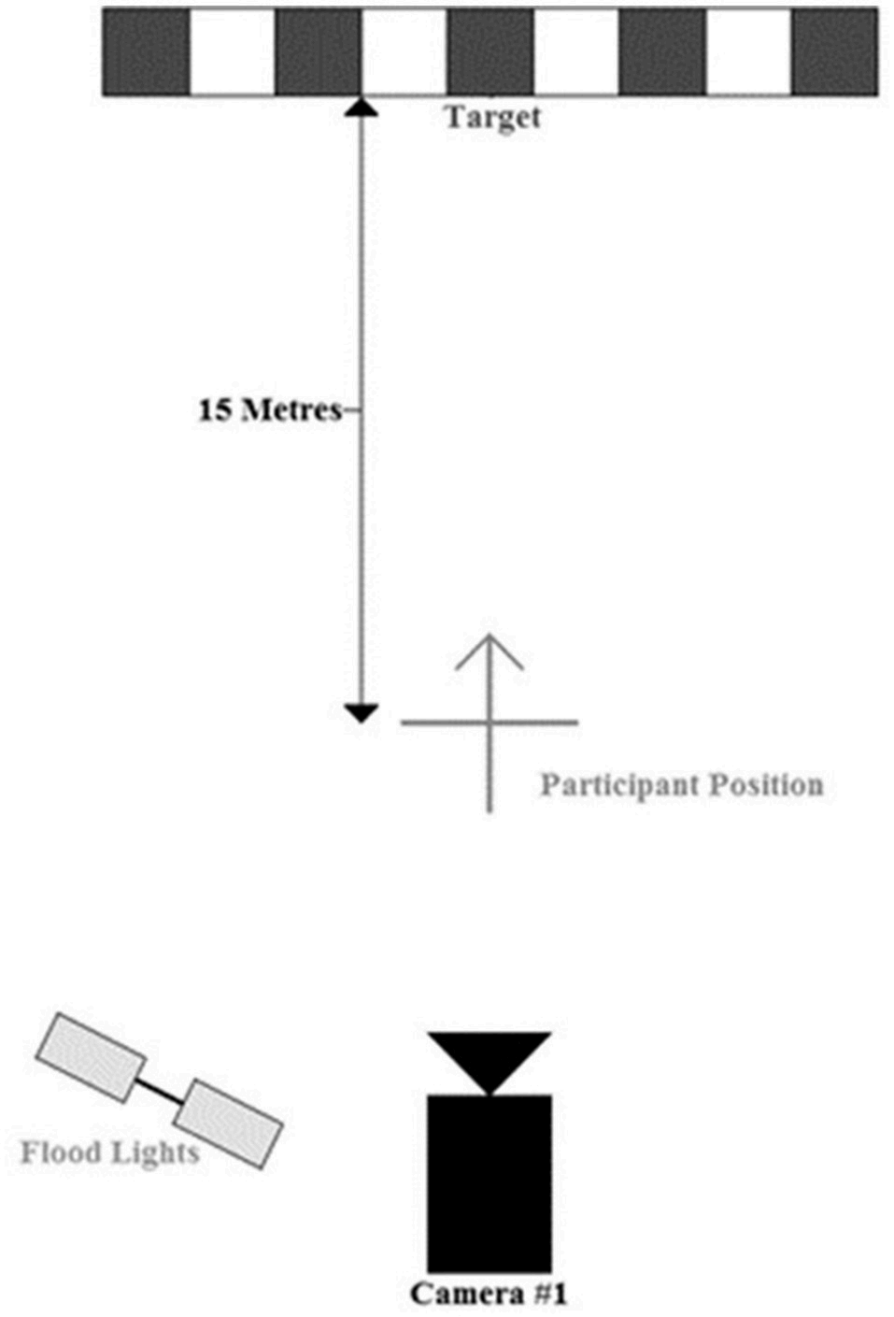
**Scoring system measured in the horizontal plan and experimental set up**.

The central target (bullseye) was 15 m away from the participant with additional scoring zones extending in 25 cm squares to the left and right of the central target up to 4 m. The scoring zones were marked by laminated signs fixed to the bottom of the wall (5 m high) with adhesive, with lines separating individual zones marked on the wall extending from floor to approximately 75 cm above the floor. Participants were asked to aim toward the bullseye and their score was based on the corresponding distance to each of the zones. That is, zone one was allocated 25 cm from the central target, zone two 50 cm, zone three 75 cm, and so on (Figure 1). Participants were able to view which zones where achieved per kick but were not given specific feedback of distance from center. Instructions relayed to participants were simply complete each trial to the best of their ability.

#### Video selection and editing

The “best” non-dominant and dominant-foot kicks were selected from the pre-test (day 1) based on each individual's highest scoring kicks with both feet, which were determined by the closest distance to the center of the target area. All individuals managed two to six kicks within 75 cm of the center zone. Each individualized training sequence consisted of up to six example clips (some repeated: VSM-m group example clips, *M*_clips_ = 3.6 ± 1.3; VSM-b group example clips, *M*_clips_ = 3.1 ± 1.4) to provide a 2-min training sequence (~20 s/kick).

All participants were led to believe they were seeing footage of their non-dominant foot. However, for the VSM-m group, the training video was a 2-min video where their best dominant-foot instep kicks from their pre-test were displayed in a mirror reversed fashion. That is, videos were horizontally flipped using Adobe Premiere Pro CS3 software (Adobe Systems Inc.). As these kicks were mirror reversed they displayed apparent opposite (i.e., non-dominant) foot kicks. The same 2-min training video was constructed for participants in the VSM-b group, however in this case, only their best non-dominant instep kicks from the pre-test were used.

#### Training and post-test

Training and post-testing occurred on the same day to ensure participants did not engage in additional training during the study. When participants arrived at the training session, they were asked to warm-up as in the pre-test by completing 10 kicks with each foot toward the target bullseye. After the warm-up, participants completed 10 non-dominant side kicks and were then instructed to watch their training video using a data projector and screen (2 × 2 m), (the physical practice only group rested for 2 min during this time). Participants were aware they were watching videos of themselves performing their best kicks on the first day, though they were not told how the footage was edited. After this, participants completed another 10 kicks with their non-dominant foot, before watching the video again. This was repeated seven times which resulted in 70 completed kicks and seven views of the video. At the completion of training, participants were given a 30–45 min rest period where they were encouraged to hydrate and relax in the testing and training lab which was followed by post-testing. The procedures for the post-test were the same as for the pre-test.

#### Interview questions

In addition to quantitative measures, the researchers sought to understand the cognitive and affective processes of participants. Moreover, we wanted to assess the awareness of the video intervention groups in reference to the video-manipulations used, as well as to probe their perceptions of training effectiveness. To this end, a short semi-structure interview (five items) was conducted after the post-test. Items one to three explored participant knowledge of perception of the effectiveness of the intervention completed. e.g., what was different about your performance in the post-test? When were you most confident with your performance? Item four gathered information on which factors were attended to by the participants e.g., what were you attending to when performing you kicking trials? While item five focused on feelings associated with their experience of the intervention.

### Analysis

Data analysis was conducted using the linear mixed model analysis sub routine of the statistical software package SPSS (Version 22) to determine significance between performances in pre- and post-tests rather than an ANOVA due to some outlying data. The main dependant variable was kick accuracy (hit or miss, constant error, absolute error, variable error) taking into account fixed (group, session, foot) and random (participant) factors. Additional variables measured and compared included movement characteristics such as ball movement time, participant movement time, and lead up steps, with significance set at *p* ≤ 0.05 (Table 1). Further, the information gathered from the semi-structured interviews elicited coded and open ended responses suggesting emergent themes that were analyzed using inductive methods.

**Table 1:** **Variable Description**.

**Variable**	**Description**
Target hit or miss	• Specific ball impact location inside or outside target area; miss left (1), hit left (2), hit center (3), hit right (4), miss right (5).
Absolute error	• Distance from target center, positive value for variation to the left and right, measured in centimeters.
Constant error	• Distance from target center, positive and negative values indicate right and left of center respectively, measured in centimeters.
Variable error	• Standard deviation measuring shot consistency, measured in centimeters.
^*^Movement time	• A reflective marker on the ankle was tracked from the frame indicating the first movement when starting from a stationary position, until frame indicating foot contact with the ball (ms).
^*^Steps	• Number of steps participant takes between commencing their movement and striking the ball.
^*^Ball movement time	• Time between participant striking the ball and the ball contacting the wall, measured in seconds.

* *These variables were measured from video footage sourced during the testing sessions*.

## Results

### Accuracy

Despite predictions with regard to accuracy, no significant main effects were indicated for group. However, analysis of *Target Hit or Miss* indicated a significant overall effect for foot *F*_(1, 1470)_ = 181.34, *p* ≤ 0.001. Referring to Table 1, each kick was assigned a value depending on the impact location with the target area. When group and session were combined, participants were more accurate with their right foot (*M* = 2.68, *SD* = 0.27) compared to their left (*M* = 3.52, *SD* = 0.27) *p* ≤ 0.001. This indicates the impact locations of balls kicked with the right foot were closer to the center because the mean value was closer to 3 (bullseye). However, there was no significant main effect for group or session, nor any significant interactions for *Target Hit or Miss*.

**Figure 2:**
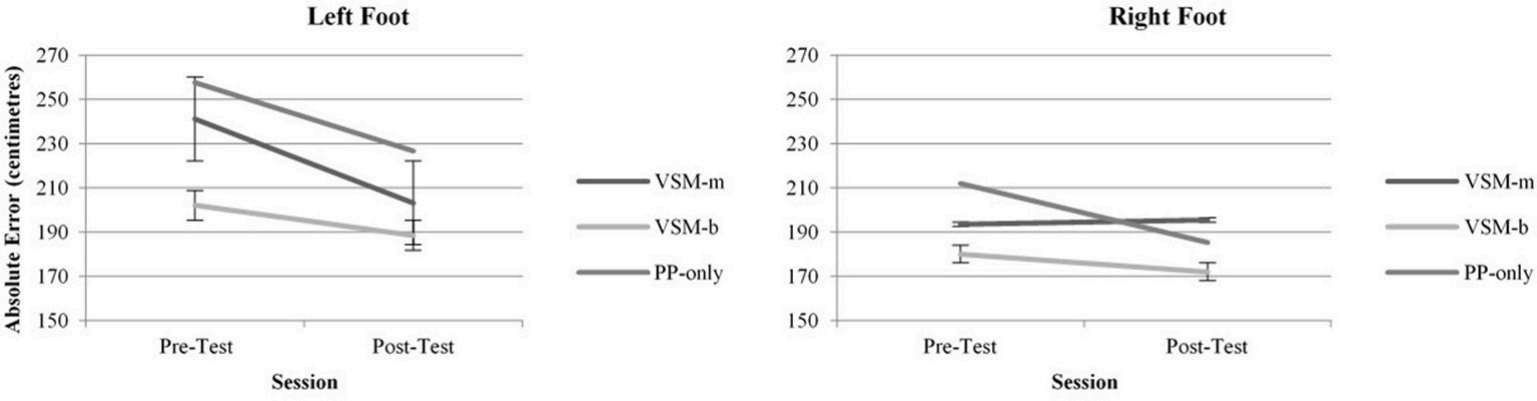
**Absolute Error for left and right feet across Pre- and Post-Tests, measured in centimeters from target center**.

Data analysis of *Absolute Error* indicated a significant overall effect for session *F*_(1, 1572)_ = 7.09, *p* = 0.008 (Figure 2). When group and foot were combined, participants were more accurate in the post-test (*M* = 195.17, *SD* = 55.57) than the pre-test (*M* = 214.3, *SD* = 55.57). Further, there was also a significant overall effect for foot *F*_(1, 1572)_ = 17.58, *p* ≤ 0.001 (Figure 2). When group and session were combined, participants were more accurate with their right foot (*M* = 189.66, *SD* = 58.47) compared to their left (*M* = 219.83, *SD* = 58.47). However, there was no significant main effect for group or any significant interactions for *Absolute Error*.

Analysis of *Constant Error* indicated a significant overall effect for session *F*_(1, 1572)_ = 5.53, *p* = 0.019. When group and foot were combined, participants were more accurate in the post-test (*M* = 11.28, *SD* = 49.53) than the pre-test (*M* = 39.17, *SD* = 49.53). Further, there was also a significant overall effect for foot *F*_(1, 1572)_ = 219.69, *p* ≤ 0.001. When group and session were combined, participants were more accurate with their right foot (*M* = −62.70, *SD* = 52.12) compared to their left (*M* = 113.15, *SD* = 52.12). However, there was no significant main effect for group or any significant interactions for *Constant Error*.

**Figure 3:**
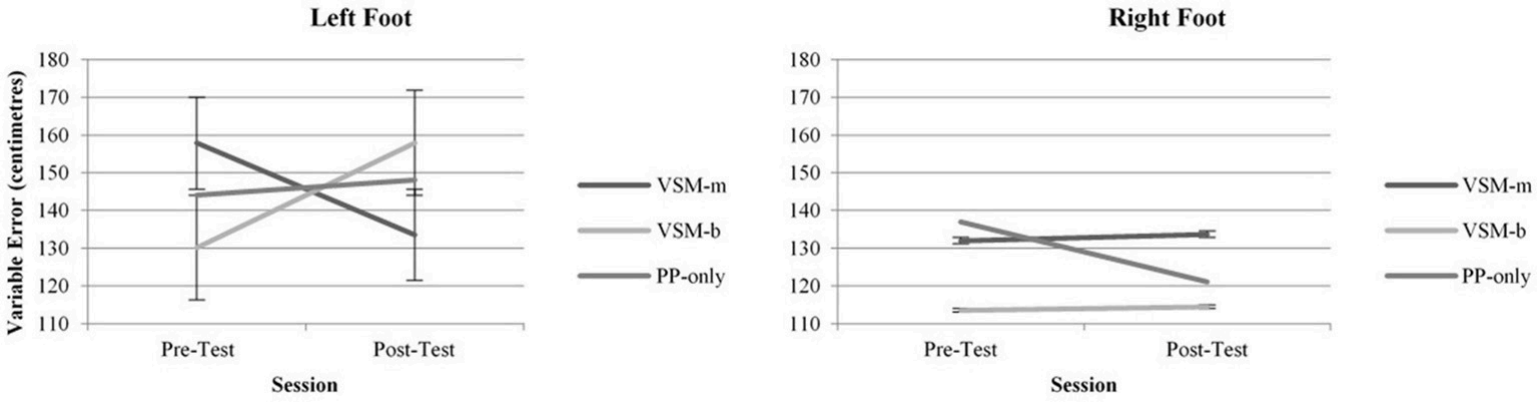
**Variable Error in left and right feet across Pre- and Post-Tests, measured in centimeters from target center**.

Analysis of *Variable Error* indicated a significant overall effect for foot *F*_(1, 1419)_ = 104.83, *p* ≤ 0.001 (Figure 3). When group and session were combined, participants were more accurate with their right foot (*M* = 125.29, *SD* = 31.19) compared to their left (*M* = 145.26, *SD* = 31.19). Further, a number of significant interactions were discovered, including group × session *F*_(2, 1419)_ = 16.53, *p* ≤ 0.001 (Table 2), group × foot *F*_(2, 1419)_ = 7.24, *p* = 0.001, and group × session × foot *F*_(2, 1419)_ = 17.92, *p* ≤ 0.001. The group × session interaction was attributable to differences in *Variable Error* in the pre-test when scores for both feet were combined, specifically Group 1 (VSM-m) and Group 2 (VSM-b) were more consistent when kicking than Group 3 (PP) (VSM-m: *M* = 17.64, *p* =.01; VSM-b: *M* = 16.79, *p* = 0.012) (Table 2). The group × foot interaction may be explained by differences between groups when sessions were combined, that is Group 1 were more consistent with their left foot on average than Group 2 (VSM-m: *M* = 27.06, *p* ≤ 0.001; VSM-b: *M* = −16.53, *p* = 0.014). The significant group × session × foot interaction was indicated because Group 1 were less consistent with their left kicks during the pre- test (VSM-m: *M* = −45.85, *p* ≤ 0.001). However, care should be taken considering these significant interactions because they do not provide any indication of intervention effectiveness.

**Table 2:** **Group × Session Interaction**.

**Variable**	**Group**					
	**VSM-m (*n* = 9)**		**VSM-b (*n* = 10)**		**PP (*n* = 9)**	
	**Pre**	**Post**	**Pre**	**Post**	**Pre**	**Post**
Target Hit or Miss (1–5)	3.69	3.42	3.59	3.29	3.50	3.61
SD	(0.36)	(0.36)	(0.36)	(0.36)	(0.36)	(0.36)
Absolute Error (cm)	241.08	203.19	202.02	188.47	257.60	226.64
SD	(64.91)	(64.91)	(64.91)	(64.91)	(64.91)	(64.91)
Constant Error (cm)	145.63	90.87	129.32	60.20	132.21	120.67
SD	(69.92)	(69.92)	(69.92)	(69.92)	(69.92)	(69.92)
Variable Error (cm)^*^	157.84	133.58	130.12	157.91	144.07	148.03
SD	(35.37)	(35.37)	(32.00)	(32.00)	(33.73)	(33.73)
Movement Time (s)^*^	1.09	1.17	1.11	1.36	1.12	1.20
SD	(0.21)	(0.21)	(0.21)	(0.21)	(0.20)	(0.20)
Steps (#)	1.60	1.83	1.64	2.00	1.63	1.72
SD	(0.37)	(0.37)	(0.38)	(0.38)	(0.38)	(0.38)
Ball Movement Time (s)^*^	3.90	4.22	3.37	3.71	4.34	4.20
SD	(0.95)	(0.95)	(0.95)	(0.95)	(0.95)	(0.95)

Average left foot values for all dependent variables for pre- and post-tests for all groups, data presented as mean (standard deviation) and

* *indicates significance*.

*# indicates “number” of Steps*.

### Movement analysis

Given the lack of significant overall effects for group with regard to accuracy in this study, a *post-hoc* analysis of participant movement was conducted from video of them performing in pre- and post-tests. Analysis of *participant movement time* indicated a significant overall effect for session *F*_(1, 1470)_ = 127.04, *p* ≤ 0.001, with all groups moving slower in their lead up to kick the ball in the post-test. The VSM-b group performed slower on average in the post-test (+272 ms), followed by the VSM-m group (+69 ms), then the PP only group (+59 ms). Slower participant movement times in the lead up to kicking the ball is further supported by a significant group × session interaction *F*_(2, 1470)_ = 35.18, *p* ≤ 0.001 (Table 2). Specifically, this interaction demonstrates Group 2 performed significantly faster in the pre-test when feet were combined (VSM-b: *M* = 0.248, *p* ≤ 0.001). Movement time in VSM groups likely slowed because participants were more conscious of their movements after watching themselves perform, and in the PP group because participants completed the post-test after physical practice meaning they could have been fatigued. Finally, while markers on the ankles were tracked in relation to movement time (Table 1) it is possible the sampling rate of 25 fps may not provide as much information as higher capture rates (e.g., 50 fps) which could be used in future studies.

Analysis of *Lead Up Steps* indicated a significant overall effect for session *F*_(1, 1470)_ = 116.92, *p* ≤ 0.001. When groups and feet were combined, participants on average took more steps in their lead up to kick the ball in the post-test (*M* = 1.81, *SD* = 0.37) than the pre-test (*M* = 1.61, *SD* = 0.37). Further, two significant interactions were indicated, including group × session *F*_(2, 1470)_ = 20.03, *p* ≤ 0.001 and group × session × Foot *F*_(2, 1470)_ = 5.66, *p* = 0.004 (Table 2). The group × session interaction was attributable to the differences between groups in the pre-test when feet were combined, specifically groups 1 and 2 took more and fewer steps on average respectively (VSM-m: *M* = 0.15, *p* =.019; VSM-b: *M* = −0.21, *p* = 0.002). The group × session × foot interaction may be explained by Group 1 performing fewer steps with their left feet in the pre test (VSM-m: *M* = 0.28, *p* = 0.002). Again, these interactions cannot be attributed to the intervention because they highlight significant pre-test differences, however participants across all groups were taking an increasing number of steps in the post-test.

Analysis of *Ball Movement Time* indicated a significant overall effect for session *F*_(1, 1470)_ = 12.04, *p* ≤ 0.001. When group and foot was combined, the ball traveled slower in the post-test (*M* = 3.76, *SD* = 0.90) than the pre-test (*M* = 3.61, *SD* = 0.90). Further, there was also a significant overall effect for foot *F*(_1, 1470)_ = 182.16, *p* ≤ 0.001. When group and session was combined, the ball traveled slower when kicked with the left foot (*M* = 3.96, *SD* = 0.90) when compared to the right (*M* = 3.41, *SD* = 0.90). Moreover, two significant interactions were indicated, including group × session *F*_(2, 1470)_ = 5.00, *p* = 0.007 and group × foot *F*_(2, 1470)_ = 7.47, *p* = 0.001 (Table 2). The group × session interaction was attributable to the differences between groups in the pre-test when feet were combined, specifically *Ball Movement Time* for groups 1 and 2 was faster and slower respectively, however neither mean was significant. The group × foot interaction was indicated because the ball movement times for Group 1 were slower when kicking with the left foot when sessions were combined (VSM-m: *M* = 0.37, *p* =.009). This suggests the average force participants imparted on the ball differed significantly in the post-test, further, each group was able to impart significantly more force onto the ball with their right foot thus resulting in shorter ball travel time.

#### Interview questions

Items from a semi-structured interview were either coded or examined for emerging themes. An overview of the results showed a number of trends including that VSM groups were more likely to perceive they had improved after video training (VSM-m 73%; VSM-b 90%) compared to the PP-only group (60%). Additional open-ended items designed to establish how participants felt about the differences between their performance in the pre and post-test elicited responses that expressed similar narratives in regard to their perception of how they felt about whether they had improved or not. Further, they were designed to determine what they thought was different about their performance after the intervention period. Responses suggested that participants were more focused, relaxed, and confident when compared to how they felt in the pre-test. Further, regardless of group, participants expressed a relatively equal level of frustration, or disappointment when they did not achieve their target, with some also stating the training was somewhat tedious in the last few rounds.

**Figure 4:**
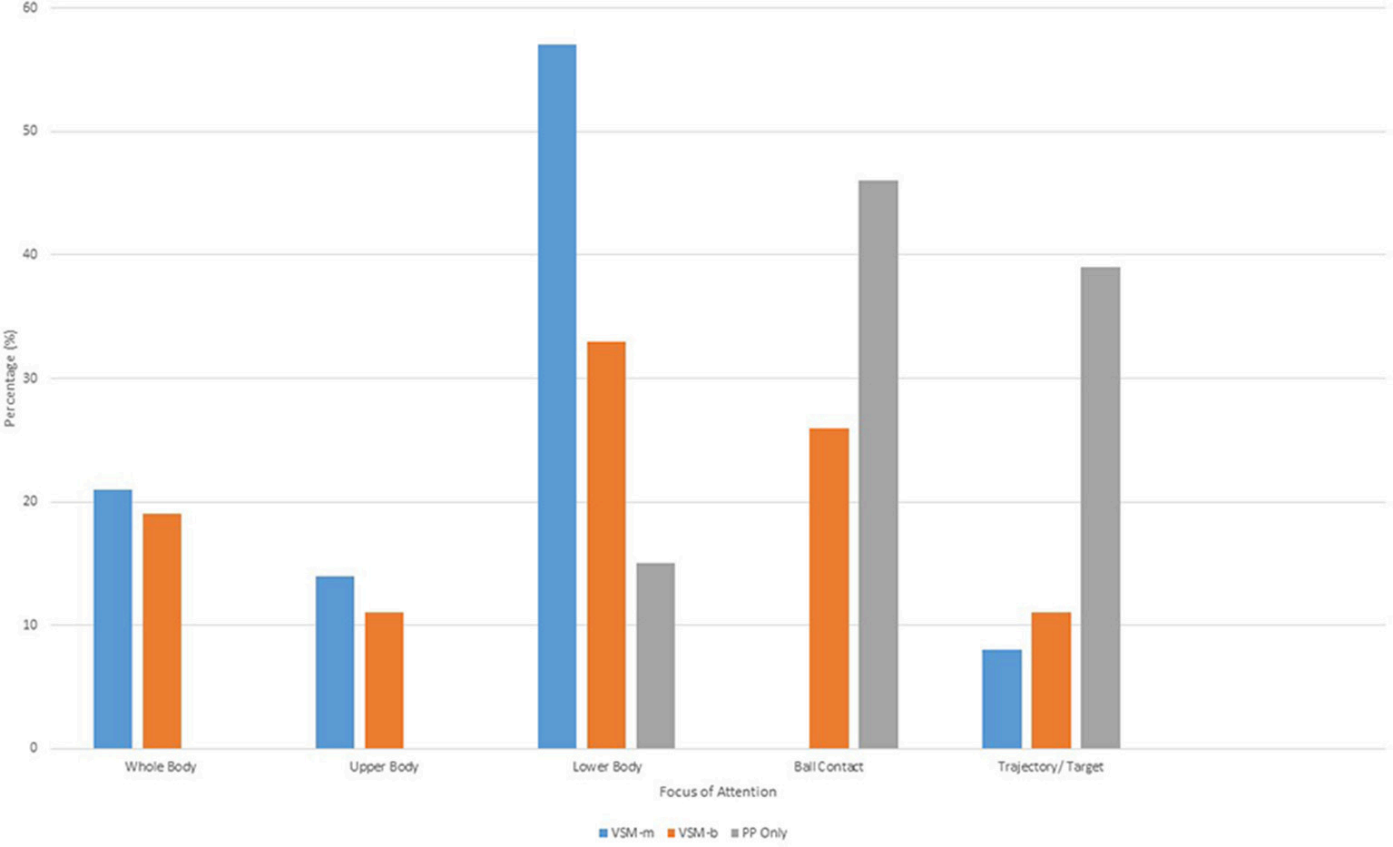
**Percentage of attentional cues afforded to various characteristics during training and testing**.

Further items explored participant attentional cues during the post-test with members of the VSM-m and VSM-b groups attending substantially more to movement variables of various body segments and coordinated systems (leg and foot, shoulder and arm, and torso), when compared to the PP-only group (Figure 4). However, the PP-only group focused more often on the target at 39% of the time compared to VSM-m and VSM-b. All groups attended to lead up steps and ball path at a similar rate (~4–8%).

**Figure 5:**
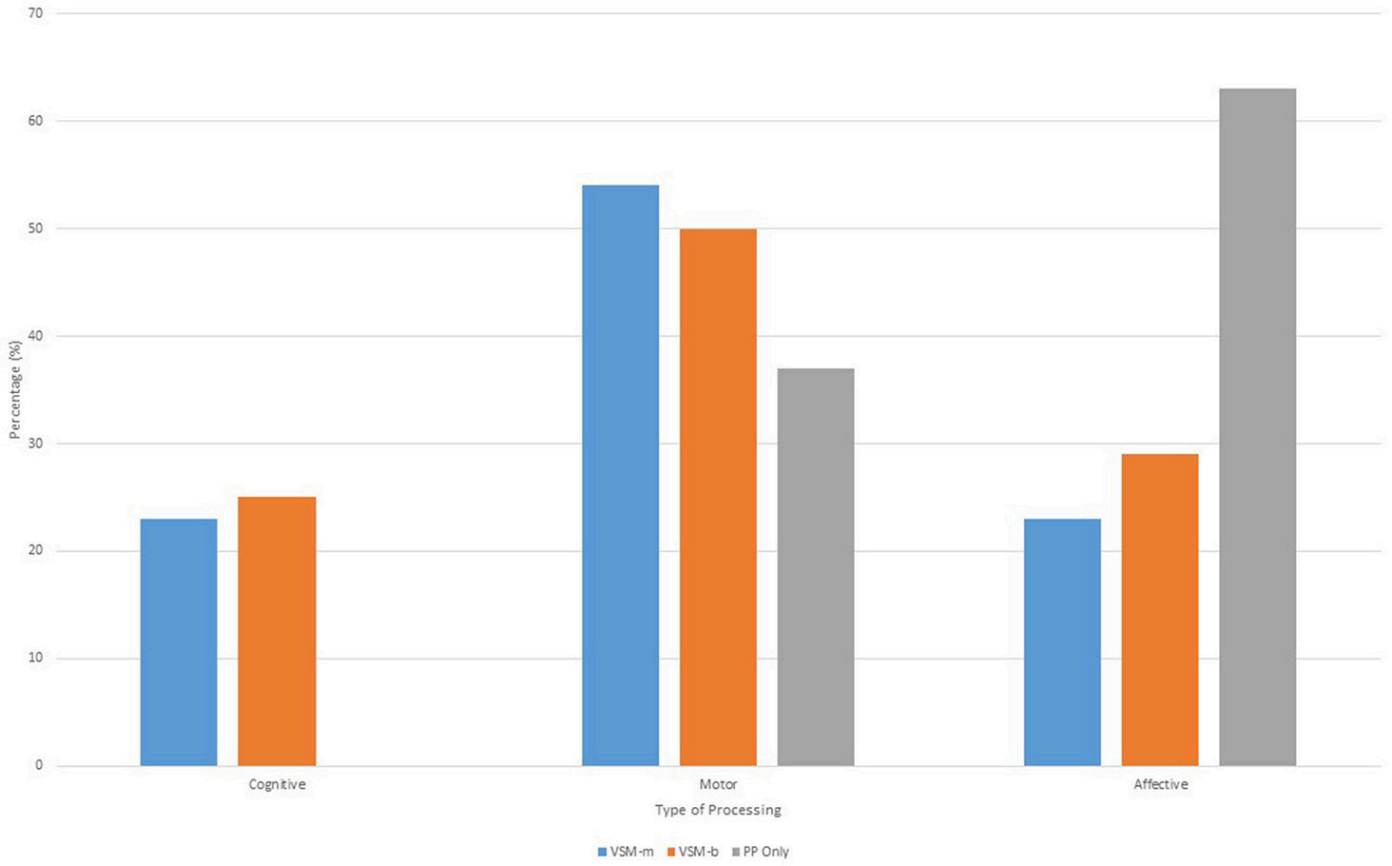
**Task analysis strategies used by each group**.

The final trend that emerged from opened-ended items revealed a number of task analysis strategies similar to those reported by Ste-Marie et al. (2011), e.g., affective, motor, and cognitive processing. Both VSM groups provided responses that can be classified as cognitive processing in nature, e.g., “I was thinking about the video, I was thinking less and was more focused,” compared to the PP-only group who did not provide any responses in this category. All groups provided a more substantial number of responses classified as motor processing, e.g., “I was more focused on the target and my hips, the video highlighted my technique, and the video influenced how I kicked,” especially the VSM groups with the PP-only group providing the least number of instances in this category. The affective processing category however elicited the strongest response from the PP-only group when referring to their performance in the post-test, with comments such as “I was less nervous and I felt training worked” (Figure 5).

## Discussion

The current study was designed to test the effectiveness of a VSM combined with physical practice (PP) intervention compared to PP-only for the improvement of a non-preferred side kicking task, and we hypothesized that VSM combined with PP would result in better kicking accuracy. We also proposed that within the two VSM conditions, participants in the “mirror reversed” VSM group would show greater improvement compared to the “best of” VSM training group. Finally, we proposed that changes in movement patterns and conscious processing would provide an insight into these changes.

An examination of the data provided varying levels of evidentiary support for the proposed hypotheses. Initial data analysis demonstrated all groups improved kicking accuracy after each training intervention based on the variables; absolute and constant error, though no VSM group was significantly better than the PP-only group. While the VSM groups did demonstrate improvement in accuracy compared to the PP-only group, with the VSM-b group showing the greatest improvement in relation to Target Hit/Miss and constant error, significance was not indicated and may be attributed to a limited sample size. The second main finding within this study relates to data gathered by qualitative methods that demonstrated varied cognitive, attentional, and affective strategies between VSM groups compared to the PP-only group.

Analysis of accuracy data in this study were not as expected and may also have been a consequence of a modified experimental set-up compared to previous studies conducted by the researchers. The first difference being previous studies employed a VSM only intervention vs. a control group and no VSM + physical practice. Moreover, training footage was captured from a frontal perspective as opposed to a posterior view, though evidence from Gonzales et al. (2015) suggests both frontal and posterior views are equally effective. Finally, previous studies used a pre, post, and retention data collection procedure which demonstrated continued improvement at the retention test as opposed to the current study which only used a pre-post design (Steel et al., 2013a,b).

An alternative explanation for these findings may be derived from studies investigating hemispheric lateralisation of motor control which have examined ipsilesional motor deficits following stroke. This research has shown that the left and right hemispheres specialize in regulating different aspects of motor control (Sainburg, 2002; Schaefer et al., 2007). The researchers suggested that the left cerebral hemisphere plays a larger role in the trajectory of the movement, while the right side plays a greater role in the final position accuracy of a movement. Therefore, in the current study the use of a mirror reversed image of the dominant foot to indicate an apparent “best of” execution of non-dominant side proficiency, may have created a discrepancy in the perception of the images compared to known movement patterns for each side. Consequently this may present a limitation for this study, though a future study involving a larger sample size is required to validate this theory.

Although overall results were not statistically significant between groups, the results using a “mixed practice” method (observational practice/physical practice), were promising and may support the findings of previous research (Ong et al., 2012). This is an important factor in numerous contexts, including sport as athletes are often exposed to training volumes and selection deadlines that make additional training ineffective and potentially injurious. Thus, mixed practice may allow for more efficient use of time with less exposure to injury. A second consideration is the use of the VSM method when athletes sustain an injury. The construction and viewing of useful VSM sequences may provide some resistance to decreases in performance thus accelerating return to form. Evidence from the current study, though in some cases not significant, demonstrated VSM groups tended to show smaller decreases in accuracy on the preferred foot performance, which was not exposed to any directed training.

The second main finding of this study related to interview responses and may provide an insight into the use of VSM interventions. Ste-Marie et al. (2011) used a qualitative measure by coding various categories including; Forethought Phase (task analysis, strategic planning, and self-motivational beliefs) and Self-reflection Phase (self-judgment). The subcategory of strategic planning is particularly important in the current study. Strategic planning includes affective, cognition, and motor factors, with participants from this study in the VSM groups providing responses suggesting that they were more likely to process information related to movement characteristics (cognitions and motor) compared to the PP-only group who attended almost exclusively to the outcome (bullseye). This suggests that observational learning provides salient visual information not afforded when performing PP only (Gonzales et al., 2015).

Interestingly, when examining the affective aspects of the strategic planning subcategory, all groups showed similar levels of frustration and embarrassment, however VSM-m and VSM-b groups found the process more tedious which may have been a reflection of the additional elements to their intervention schedule. All groups had positive affective perceptions to their training schedule (relaxed, confident) however the VSM-b group displayed the greatest variety of positivity toward the intervention, often using terms such as relaxed, good, surprised, excited, improved, focused, confident, and competitive when compared to their peers. Like previous VSM studies this provides evidence that even when movement changes may not be significant, there are substantial improvements in the perception of one's movement ability (Rymal et al., 2010). Future studies should utilize qualitative methods such as interviews to gather greater insight into these factors.

The current study also differed in design, where a pre, post, retention was used previously, and highlighted the VSM method continued to aid improvement past the cessation of the intervention period. It is possible the retention period acts as consolidation period for learning in this case. The decision not to include a retention test was based on recruitment difficulties whereby asking participants to return for a third visit tended to deter them. It is possible however the short period of time between the intervention and post-testing did not allow time for movement learning to consolidate. Therefore, a further study should include three test occasions with the possibility of a greater period of time between the intervention and the post-test.

Finally, analysis of movement variables demonstrated no group-related differences related to kick accuracy, movement time, or lead up steps, however there were some significant overall effects for session. Participants took longer to move in the post-test and also increased the number of steps for non-dominant foot kicks following the video training. These types of changes are typical when learning new skills where individuals may take longer to execute the movement due to increased cognitive engagement with the new or modified movement pattern. Further, additional lead up steps may have been a reflection upon the video clips selected for training, where participants associated the number of steps with successful execution.

## Conclusions

Results from the current study suggest that VSM when combined with physical practice is not significantly more effective than physical practice alone, however positive trends toward increased kick accuracy were observed. Responses gained through qualitative measures however did suggest VSM methods provided participants with a greater sense of improvement and also directed participant attention to movement variables rather than target outcome. Further research is required to determine the factors that contribute to the consistent application of this method for the improvement of movement skills, and may consider footage view point, the duration of intervention periods, video sampling rate and type of skill examined.

## Author contributions

KS: performed the following roles: design, implementation, data collection and analysis, and primary writer. EE: data analysis and secondary writer.

### Conflict of interest statement

The authors declare that the research was conducted in the absence of any commercial or financial relationships that could be construed as a potential conflict of interest.
